# Factors affecting Pakistani young adults’ intentions to uptake COVID‐19 vaccination: An extension of the theory of planned behavior

**DOI:** 10.1002/brb3.2370

**Published:** 2021-09-20

**Authors:** Irfan Ullah, Chung‐Ying Lin, Najma Iqbal Malik, Tzu‐Yi Wu, Marzieh Araban, Mark D. Griffiths, Amir H. Pakpour

**Affiliations:** ^1^ Kabir Medical College Gandhara University Peshawar Pakistan; ^2^ Institute of Allied Health Sciences, College of Medicine National Cheng Kung University Tainan Taiwan; ^3^ Department of Occupational Therapy, College of Medicine National Cheng Kung University Tainan Taiwan; ^4^ Department of Public Health, National Cheng Kung University Hospital, College of Medicine National Cheng Kung University Tainan Taiwan; ^5^ Department of Psychology University of Sargodha Sargodha Punjab Pakistan; ^6^ Department of Occupational Therapy, College of Medical and Health Science Asia University Taichung Taiwan; ^7^ Department of Health Education and Promotion, Public Health School Ahvaz Jundishapur University of Medical Sciences Ahvaz Iran; ^8^ International Gaming Research Unit, Psychology Department Nottingham Trent University Nottingham UK; ^9^ Department of Nursing, School of Health and Welfare Jönköping University Jönköping Sweden; ^10^ Social Determinants of Health Research Center, Research Institute for Prevention of Non‐Communicable Diseases Qazvin University of Medical Sciences Qazvin Iran

**Keywords:** COVID‐19 vaccination, fear of COVID‐19, perceived infectability, Protection Motivation Theory, Theory of Planned Behavior

## Abstract

**Introduction:**

Aside from personal beliefs, young adults’ intention to uptake the COVID‐19 vaccine can be influenced by their fear of COVID‐19 and perceived infectability of COVID‐19. The present study incorporated fear of COVID‐19 and perceived infectability with the theory of planned behavior (TPB) to form an expanded TPB to analyze factors affecting Pakistani young adults’ intentions to uptake the COVID‐vaccine in Pakistan.

**Methods:**

A cross‐sectional study was conducted and recruited participants from Pakistani social media users. The proposed extended TPB model was examined by using structural equation modeling.

**Results:**

A total of 1034 individuals replied to the survey. The three factors of the original theory of planned behavior and the fear of COVID‐19 were positively related to their intention to uptake COVID‐19 vaccination (*r *= 0.25‐0.66). Moreover, the perceived infectability positively influenced the three theories of planned behavioral factors and the fear of COVID‐19 (*r *= 0.27‐0.60), also affecting the participants’ intentions to uptake COVID‐19 vaccination.

**Conclusions:**

Perceived infectability was positively related to the participants’ intentions to uptake COVID‐19 vaccination, and perceived behavioral control was the strongest mediator. More evidence‐based information concerning treatments and COVID‐19 vaccination are needed to encourage individuals to uptake the vaccine.

## 1 INTRODUCTION

Coronavirus disease 2019 (COVID‐19) is an emerging, rapidly evolving disease worldwide (World Health Organization, 2021b). At the time of writing (August 3, 2021), the World Health Organization reported that 199.89 million people had been infected with COVID‐19 and over 4.25 million had died since the start of the pandemic (World Health Organization, 2021a). In Pakistan (where the present study was carried out), the cumulative cases of COVID‐19 were more than 1.043 million, and over 25,500 deaths according to the report of Government of Pakistan by August 2021. Meanwhile, over four million Pakistanis have been vaccinated equating to approximately 30% of the population. Moreover, the pandemic has resulted in severe economic and social disruption.

The development of COVID‐19 vaccines has brought possible protection against the disease. To date, three COVID‐19 vaccines have been authorized the use in certain countries, but only *AstraZeneca* has published results about efficacy and safety of the vaccine in the peer‐reviewed literature (World Health Organization, 2020). No matter how long the vaccine provides protection, clinical trials demonstrated that vaccination was effective in reducing the rate at which the disease spreads. However, not all individuals are willing to be vaccinated due to misleading information, complacency, the convenience of vaccine obtainment, and lack of confidence concerning vaccine safety (Fisher et al., 2020; Mercadante & Law, 2020). The refusal to uptake COVID‐19 vaccines may decrease the effectiveness of disease control and could threaten public health.

Interventions that use theory to develop healthy behaviors provide a valuable framework for accumulating evidence and may provide greater changes in health‐related behavior (Prestwich et al., 2015). The Theory of Planned Behavior (TPB) has been successful in predicting several health behaviors (Corace et al., 2016; Lin et al., 2018, 2017, 2016; Schmid et al., 2017; Strong et al., 2018; Xiao & Wong, 2020). The TPB was adapted from the Theory of Reasoned Action to understand and predict whether an individual would take a specific action (Ajzen, 1991). The TPB presumes that an individual's behavioral intention is affected by three factors: attitude, subjective norm, and perceived behavioral control. Behavioral intention further affects the individual's behaviors (Icek Ajzen, 1991). In the case of COVID‐19 vaccination uptake, attitude means the individual's positive or negative judgments to uptake COVID‐19 vaccines. Subjective norms refer to individual's belief about the prevalence of vaccine uptake or the social pressures that individuals feel when deciding whether to vaccinate. For example, the stress of whether their significant others (e.g., parents, spouses, friends, and colleagues) think they should be vaccinated. Perceived behavioral control indicates the individual's perceptions of the degree of external resources and self‐efficacy, such as time, money, and the opportunity to get COVID‐19 vaccines. The aforementioned three factors would affect the degree or tendency of the individual's behavior intention toward vaccine uptake. Overall, the TPB claims that, when the behavior is volitional control, attitude, subjective norm, and perceived behavioral control mutually determine an individual's behavioral intention, and perceived behavioral control can directly affect the behavior (Ajzen, 1985; Xiao & Wong, 2020).

Moreover, the TPB can be supplemented by fear of disease and perceived vulnerability in Protection Motivation Theory (PMT), which improves researchers’ understanding of an individual's intention to vaccinate against COVID‐19 (Yahaghi et al., 2021). The PMT is a health psychological theory and was developed to explain the cognitive mediation process of behavioral change from the perspective of threat and coping appraisal (Rogers, 1975; Rogers & Prentice‐Dunn, 1997). The PMT's threat appraisal component contains two major elements: perceived severity and perceived vulnerability, which are affected by fear of disease. The coping appraisal comprises the following: response efficacy (i.e., the judgments whether accomplish an action can decrease the threat), self‐efficacy (i.e., the confidence that an individual has abilities to take action to protect themselves), and response cost (i.e., the cost related to take action to remove the threat) (Norman et al., 2005). In this case, fear of disease (i.e., COVID‐19) can be reflected by how individuals consider severity of having COVID‐19 and the ability to cope with it. Perceived vulnerability means the perceived infectability of COVID‐19.

**FIGURE 1:**
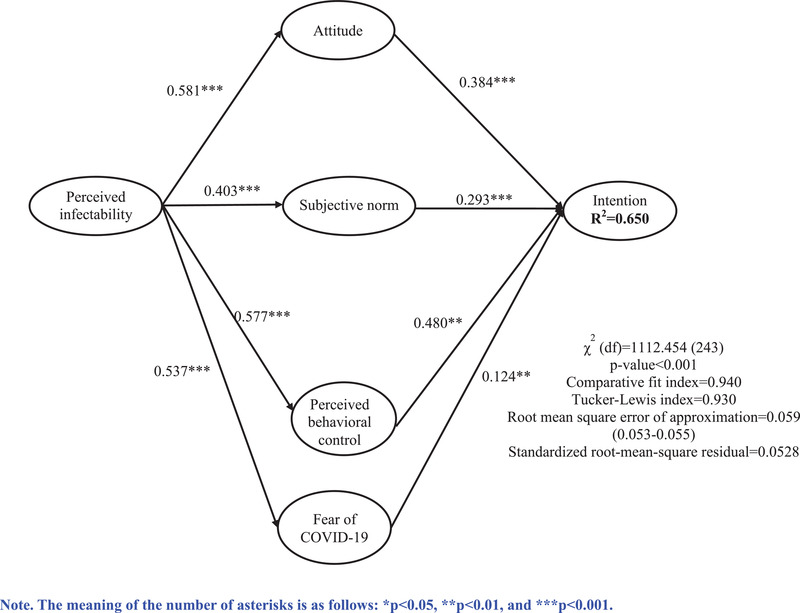
The structural equation modeling results of the proposed model

The present study supplemented the PMT elements of fear and perceived infectability to the TPB for maximizing the efficiency in explaining individual's intention to be vaccinated for COVID‐19 infection. Some studies have found that perceived vulnerability increases individuals’ positive attitude toward the behavior to prevent the threat (Pang, Tan, & Lau, 2021), and trigger their review of the environment, resources, and self‐efficacy to take protective action (Najafi et al., 2017). Moreover, when individuals feel more vulnerable, they may tend to accept significant others’ opinion in protecting themselves. Therefore, it was hypothesized that perceived infectability would affect the intention to uptake the COVID‐19 vaccine through individual attitudes, subjective norms, perceived behavior control, and fear of COVID‐19 (see Figure 1). The proposed hypotheses were that:

1. Perceived infectability will have a positive influence on Pakistani young adults’ attitude to uptake the COVID‐19 vaccine, and their attitude might positively affect their intention to uptake the COVID‐19 vaccine.
2. Perceived infectability will have a positive influence on Pakistani young adults’ subjective norm to uptake the COVID‐19 vaccine, and subjective norm could positively affect their intention to uptake the COVID‐19 vaccine.
3. Perceived infectability will have a positive influence on Pakistani young adults’ perceived behavioral control to uptake the COVID‐19 vaccine, and perceived behavioral control could positively affect their intention to uptake the COVID‐19 vaccine.
4. Perceived infectability will be positively associated with Pakistani young adults’ fear of COVID‐19 to uptake the COVID‐19 vaccine, and fear of COVID‐19 could positively affect their intention to uptake the COVID‐19 vaccine.

## 2 MATERIALS AND METHODS

### 2.1 Participants and procedure

A cross‐sectional study was conducted among social media users in Pakistan from 25 December 2020 to 8 January 2021. The study was approved by the Institutional Review Board (IRB) at the University of Sargodha, Pakistan (Ref: SU/PSY/889‐S). All items in the survey were set as required items in *Google Forms* to avoid missing data. In short, participants were unable to submit the responses on the survey unless all questions had been answered. The inclusion criteria of the present study were being (i) Pakistani (permanent resident), (ii) over the age of 15 years, and (iii) able to understand Urdu or English. Informed consent for the survey was ensured and a valid email address was requested from each participant. All data were checked for similarity to ensure each participant only completed the survey once.

### 2.2 Instruments

2.2.1 *Measures on Theory of Planned Behavior (TPB)*. Items on *Attitude* (six items; sample item: For me, getting the COVID‐19 vaccination would be… extremely bad [score 1] to extremely good [score 5]), *Subjective norms* (two items; sample item: Most individuals who are important to me would want me to get vaccinated, strongly disagree [score 1] to strongly agree [score 5]), *Perceived behavioral control* (two items; sample item: Whether or not I get COVID‐19 vaccination is completely up to me, strongly disagree [score 1] to strongly agree [score 5]), and *Intention* (two items; sample item: I am willing to get COVID‐19 vaccination, strongly disagree [score 1] to strongly agree [score 5]) were designed in the present study according to Ajzen's guidelines (Ajzen & Schmidt, 2020). Moreover, the measures concerning TPB in the present study had satisfactory psychometric properties: Cronbach's α = 0.886 for attitude, 0.833 for subjective norms, 0.776 for perceived behavioral control, and 0.888 for intention; composite reliability (CR) = 0.893 for attitude, 0.841 for subjective norms, 0.776 for perceived behavioral control, and 0.888 for intention; average variance extracted (AVE) = 0.586 for attitude, 0.728 for subjective norms, 0.540 for perceived behavioral control, and 0.746 for intention.

2.2.2 *Measures on perceived infectability*. Seven perceived infectability items from the Perceived Vulnerability to Disease Questionnaire were used to assess perceived infectability (sample item: If a COVID‐19 patient is “going around,” I will get it, strongly disagree [score 1] to strongly agree [score 5]) (Díaz et al., 2016). The measures on perceived infectability in the present study had satisfactory psychometric properties: α = 0.760; CR = 0.876; and AVE = 0.504.

2.2.3 *Measures on Fear of COVID‐19*. The Fear of COVID‐19 Scale (FCV‐19S; Ahorsu et al., 2020) was used to assess fear of COVID‐19 (sample item: I am most afraid of COVID‐19, strongly disagree [score 1] to strongly agree [score 5]). The FCV‐19S was developed using Protection Motivation Theory (Rogers, 1975). The measures on fear of COVID‐19 in the present study had satisfactory psychometric properties: α = 0.899; CR = 0.892; and AVE = 0.544.

### 2.3 Data analysis

The participants’ characteristics were firstly analyzed using descriptive statistics including means and frequencies. Pearson correlations were used to examine the associations between every two tested constructs proposed in the model. Following this, structural equation modeling (SEM) was carried out to verify the construct validity of the measurements used in the present study and to examine the proposed model. For construct validity, confirmatory factor analysis (CFA) was applied to ensure that the items belonged to the construct as expected. Reliability coefficients, factor loadings, and average variance extracted statistics of each domain of all measurement tools were calculated. In this model, all the constructs were formed as latent using the items described in the *Instruments* section. The estimator used for the SEM was maximum likelihood estimation with the missing values tackled using full information maximum‐likelihood estimation. Moreover, the mediated effects of attitude, subjective norms, perceived behavioral control, and the fear of COVID‐19 in the association between perceived infectability and intention to vaccination uptake were assessed using the bootstrapping method. More specifically, the bias corrected percentile with 5000 bootstrapping samples was used, and the 95% confidence interval (CI) among the 5000 bootstrapping samples was calculated. When the 95% CI does not cover 0, the mediated effects are significant. The entire SEM model was evaluated using commonly used fit indices: comparative fit index (CFI) > 0.95, Tucker‐Lewis index (TLI) > 0.95, root mean square error of approximation (RMSEA) < 0.08, and standardized root‐mean‐square residual < 0.08. SEM analysis was then further performed stratified by gender to verify if there was a gender bias. The statistical analyses were carried out using SPSS, except for the SEM using AMOS.

## 3 RESULTS

The study sample comprised more females (n = 743; 71.9%) than males (n = 291; 28.1%) and had a relatively young age (mean = 22.17 years; SD = 6.36). The majority of the participants had a degree (23.5% postgraduate and 48.0% undergraduate). Less than one‐fifth of the participants were currently married (13.3%). Very few participants had been infected by the COVID‐19 (3.7%) and even fewer participants had been hospitalized due to COVID‐19 (1.5%) (Table 1).

**TABLE 1:** The demographic characteristics of the participants included in this study

	Mean ± SD or N (%)
***Age***	22.17 ± 6.36
***Gender***	
Male	291 (28.1%)
Female	743 (71.9%)
***Educational status***	
Univesity (postgraduate)	243 (23.5%)
University (undergraduate)	496 (48.0%)
Secondary school	186 (18.0%)
Primary school	55 (5.3%)
***Marital status***	
Married	138 (13.3%)
Single	889 (86.0%)
Divorced/widowed	7 (0.7%)
***Past Covid‐19 hospitalization***	
Yes	16 (1.5%)
***Past Covid‐19 infection***	
Yes	38 (3.7%)
***Current smoking status***	
Yes	41 (4.0%)

Table 2 demonstrates that the tested constructs were significantly associated with each other with the magnitude ranging between small and large effects (*r *= 0.196 to 0.700). The means and SDs are also reported in Table 2.

**TABLE 2:** The correlations of theory planed behaviour variables

SD	Mean	6	5	4	3	2	1	
0.87	3.81	0.196**	0.271**	0.413**	0.437**	0.528**	1	**1. Attitude**
1.14	3.22	0.264**	0.525**	0.664**	0.700**	1	–	**2. Subjective nomrs**
1.03	3.16	0.272**	0.603**	0.627**	1	–	–	**3. Percevied behavioral control**
1.09	2.99	0.251**	0.578**	1	–	–	–	**4. Intention**
0.83	2.92	0.299**	1	–	–	–	–	**5. Percevied vulnerability**
5.55	15.18	1	–	–	–	–	–	**6. Fear of COVID‐19**

** *p* < 0.01.

In the results of CFA, all factor loadings of the items used in the proposed model were strong (range between 0.538 and 0.846 for attitude; 0.769 and 0.930 for subjective norms; 0.724 and 0.746 for perceived behavioral control; 0.860 and 0.868 for intention; 0.634 and 0.824 for perceived infectability; and 0.610 and 0.799 for fear of COVID‐19) (Table 3). The reliability coefficients (α) ranged from 0.776 to 0.899. The values of composite reliability ranged from 0.700 to 0.893. The values of average variance extracted statistics ranged from 0.504 to 0.746. The proposed model (Figure 1) showed that it fitted well with the data (CFI = 0.940, TLI = .930, RMSEA = 0.059 with 90% CI between 0.055 and 0.053, and SRMR = 0.0528). Moreover, all the proposed paths had their coefficients significant with the largest association shown between perceived behavioral control and intention (standardized coefficient = 0.480) and the weakest association shown between fear of COVID‐19 and intention (standardized coefficient = 0.124). Furthermore, Table 4 shows that the mediated effects were significant (standardized coefficient = 0.305; 95% CI = 0.487‐0.734; *p *< 0.001), which indicated that attitude, subjective norms, perceived behavioral control, and fear of COVID‐19 mediated the association between perceived infectability and intention to vaccination uptake. The results of the SEM analysis stratified by gender were similar to the model including all participants (Supporting information Figure S1).

**TABLE 3:** Items for study measures with descriptive statistics, confirmatory factor analysis, reliability coefficients, factor loadings, and average variance extracted statistics (AVE)

Construct	Measurement item	λ	α	CR	AVE
Attitude	*For me, getting the COVID‐19 vaccination would be …*		0.886	0.893	0.586
	extremely bad (1)/extremely good (5)	0.750			
	extremely undesirable (1)/extremely desirable (5)	0.796			
	extremely unimportant (1)/extremely important (5)	0.846			
	extremely useless (1)/extremely useful (5)	0.823			
	extremely unfavorable (1)/extremely favorable (5)	0.800			
	extremely harmful (1)/extremely beneficial (5)	0.538			
Subjective norms	*Most people who are important to me would…*		0.833	0.841	0.728
	want me to get COVID‐19 vaccination	0.769			
	think I should get COVID‐19 vaccination	0.930			
Perceived behavioral control			0.776	0.700	0.540
	Whether or not I get COVID‐19 vaccination is completely up to me.	0.724			
	I have resources, time and opportunities to get COVID‐19 vaccination.	0.746			
Intention	I am willing to get COVID‐19 vaccination.	0.860	0.888	0.854	0.746
	I want to get COVID‐19 vaccination.	0.868			
Perceived infectability			0.760	0.876	0.504
	If a COVID‐19 patient is “going around,” I will get it.	0.690			
	My past experiences make me believe I am not likely to get sick even when my friends are sick.	0.667			
	I have a history of susceptibility to infectious diseases	0.634			
	In general, I am very susceptible to colds, flu, COVID‐19 and other infectious diseases.	0.696			
	I am more likely than the people around me to catch COVID‐19.	0.698			
	I am unlikely to catch a cold, flu, COVID‐19 or other illness, even if it is “going around.”	0.732			
	My immune system protects me from most illnesses that other people get.	0.824			
Fear of COVID‐19	I am most afraid of coronavirus‐19	0.718	0.899	0.892	0.544
	It makes me uncomfortable to think about coronavirus‐19	0.610			
	My hands become clammy when I think about coronavirus‐19	0.799			
	I am afraid of losing my life because of coronavirus‐19.	0.786			
	When watching news and stories about coronavirus‐19 on social media, I become nervous or anxious	0.768			
	I cannot sleep because I'm worrying about getting coronavirus‐19	0.743			
	My heart races or palpitates when I think about getting coronavirus‐19	0.727			

*Note*. Λ, Standardized factor loading from structural equation model; α, Cronbach's alpha reliability coefficient; ω, McDonald's omega reliability coefficient; CR, Composite reliability; AVE, Average variance extracted from structural equation model.

**TABLE 4:** The Bias‐Corrected Bootstrap Test for mediated effects through attitude, subjective norm, perceived behavioral control and Perceived vulnerability

Independent variable	Std. coefficient	Coefficient	SE	95% CI	*p*‐value
**Perceived infectability**	**0.305**	**0.630**	**0.061**	**0.487‐0.734**	**<.001**

Std. coefficient , Standardized coefficient.

## 4 DISCUSSION

The present study is the first in Pakistan to investigate the factors determining young adults’ intention to uptake the COVID‐19 vaccine using TPB and PMT. A total of 3.7% of the participants had COVID‐19 infection in the previous year. The results suggested that perceived behavior control, attitude, perceived infectability, and fear of COVID‐19 had significant impacts on the intention to get COVID‐19 vaccination among young Pakistani adults. Moreover, perceived infectability was related to the three behavioral factors and fear of COVID‐19 in a low to moderate manner.

The three behavioral factors of TPB, perceived infectability, and fear of COVID‐19 explained more than 50% of the variance in COVID‐19 vaccination uptake intention, indicating that the combination of TPB and PMT can effectively explain young Pakistani adults’ intention to get COVID‐19 vaccination. Furthermore, the expanded model showed significant paths from perceived infectability to the three behavioral factors of TPB and fear of COVID‐19, and then to intention. A previous study appears to support the findings of the present study. It found that some individuals felt that others had a higher risk of getting COVID‐19 infection and were more in need of COVID‐19 vaccination than themselves, so that they decided not to get vaccinated (Rieger, 2021). Surprisingly, individual's fear of COVID‐19 appears to be less influential than the other three factors. A previous study also found that trusting the safety of the vaccine was a stronger factor in predicting COVID‐19 vaccination intentions than the fear of COVID‐19 (Karlsson et al., 2021). In other words, an individual's attitude to get vaccine and perceived behavioral control were more influential.

The results showed that perceived infectability was related to participant attitude, subjective norms, perceived behavioral control, and fear of COVID‐19. The fear of disease comes from perceived threats (i.e., perceived susceptibility and perceived severity), and it has been verified as a crucial factor in taking action (Corrigan et al., 2014; Weston et al., 2020). The individual's attitude, and the judgment to vaccinate or not, can reflect the degree of perceived infectability. Perceived infectability also activates the individual's perceived behavioral control in reviewing the capability and resources to overcome the disease. Moreover, the effects of the subjective norm may be elevated by the increase of perceived infectability. For example, individuals might follow their significant others’ opinion to keep distance from the community to reduce the spread of COVID‐19 when they feel more threatened (i.e., higher perceived infectability) and do not know what to do (Alijanzadeh & Harati, 2021). In short, the perceived infectability influences the intention to get COVID‐19 vaccinated through the four mediators: participant’ attitude, subjective norms, perceived behavioral control, and fear of COVID‐19.

The study found that perceived behavioral control was the strongest mediator in intention to be vaccinated, and the fear of COVID‐19 was the least strong. This situation was consistent with previous studies concerning applications of behavioral change. In the TPB, perceived behavioral control comprises perceived self‐efficacy and perceived controllability (Ajzen, 2002). Self‐efficacy has been found to be an important predictor in the application of health behavioral change (Kelly et al., 1991; Strecher et al., 1986). Without self‐efficacy, the explained variance of the entire model was reduced. In addition, fear of disease may appear to lead to preventive actions at first glance, but a fear strategy may not be effective and may even be counterproductive (Shen, 2011). For example, warnings that smoking can cause lung cancer may increase anxiety and make individuals smoke more often. Therefore, some scholars have begun to call on health authorities not to use fear appeals in the health communication of COVID‐19 prevention (Stolow et al., 2020).

The present study has two key implications. First, subjective norm was quite related to intention of COVID‐19 vaccination uptake, indicating that health providers need to provide more evidence concerning safety and effectiveness of COVID‐19 vaccines and emphasize the benefits to individuals and the population. Therefore, the subjective norm would lead to positive reasons to vaccinate. Second, the perceived infectability had a significant impact on individuals’ vaccination intention, which means that the readable information concerning the incidence and prevalence of the COVID‐19 are necessary to help individuals to link the information to themselves.

The present study has three major limitations. First, the participants of this study were recruited via the internet, and they were younger than the mean age of the Pakistan population. Second, the proportion of female participants was much higher than the proportion of men. However, the results showed that the loadings of SEM were similar in both genders. Therefore, the gender imbalance might not be a critical issue in relation to generalization. Third, the participants in the present study were younger and more educated than the average of the national Pakistani population. Therefore, the results cannot be generalized to older and lower educated populations in Pakistan. These limitations may hamper the representativeness and interpretation of the study's results.

## 5 CONCLUSIONS

Perceived infectability was positively related to the participants’ intentions to uptake the COVID‐19 vaccine, and perceived behavioral control was the strongest mediator. More evidence‐based information concerning the treatments and COVID‐19 vaccination are needed to encourage individuals to uptake the vaccine.

## CONFLICT OF INTEREST

The authors declare that they have no conflict of interest.

### TRANSPARENT PEER REVIEW

The peer review history for this article is available at https://publons.com/publon/10.1002/brb3.2370

## Supporting information



Supporting Information

## Data Availability

The data that support the findings of this study are available from the corresponding author upon reasonable request.
