# Supplementary material for: Factors affecting Pakistani young adults’ intentions to uptake COVID‐19 vaccination: An extension of the theory of planned behavior
Source: Brain Behav. 2021 Sep 20;11(11):e2370. doi: 10.1002/brb3.2370 (PMC8613438; doi:10.1002/brb3.2370)
Supplement: Supplementary file 1 — Supporting Information [file BRB3-11-e2370-s001.docx]

**The Bias-Corrected Bootstrap Test for mediated effects through attitude, subjective norm, perceived behavioral control and Perceived vulnerability for males**

| **Independent variable** | **Std. coefficient** | **Coefficient** | **SE** | **95%CI** | **p-value** |
| --- | --- | --- | --- | --- | --- |
|  |  |  |  |  |  |
| **Perceived infectability** | **0.259** | **0.437** | **0.100** | **0.267-0.234** | **<0.001** |

**Std. coefficient=Standardized coefficient**

Subjective norm

Perceived
infectability

Intention

**R^2^=0.650**

Attitude

Perceived behavioral control

χ^2^ (df)=554.526 (243)

p-value<0.001

Comparative fit index=0.923

Tucker-Lewis index=0.908

Root mean square error of approximation=0.066 (0.059-0.074)

Standardized root-mean-square residual=0.0631

0.379***

0.667***

0.117**

0.536***

0.330***

0.422***

Fear of COVID-19

0.368***

0.213**

**Note. The meaning of the number of asterisks is as follows: *p<0.05, **p<0.01, and ***p<0.001.**

**The Bias-Corrected Bootstrap Test for mediated effects through attitude, subjective norm, perceived behavioral control and Perceived vulnerability for females**

| **Independent variable** | **Std. coefficient** | **Coefficient** | **SE** | **95%CI** | **p-value** |
| --- | --- | --- | --- | --- | --- |
|  |  |  |  |  |  |
| **Perceived infectability** | **0.315** | **0.664** | **0.035** | **0.520-0.848** | **<0.001** |

**Std. coefficient=Standardized coefficient**

Subjective norm

Perceived
infectability

Intention

**R^2^=0.649**

Attitude

Perceived behavioral control

χ^2^ (df)=793.395 (243)

p-value<0.001

Comparative fit index=0.951

Tucker-Lewis index=0.941

Root mean square error of approximation=0.055 (0.051-0.060)

Standardized root-mean-square residual=0.0528

0.354***

0.638***

0.174**

0.463**

0.454***

0.374***

Fear of COVID-19

0.406***

0.155**

**Note. The meaning of the number of asterisks is as follows: *p<0.05, **p<0.01, and ***p<0.001.**
